# DNA hypomethylation drives changes in *MAGE-A* gene expression resulting in alteration of proliferative status of cells

**DOI:** 10.1186/s41021-020-00162-2

**Published:** 2020-07-30

**Authors:** Ashley Colemon, Taylor M. Harris, Saumya Ramanathan

**Affiliations:** 1 Fisk-Vanderbilt Masters-to-PhD Bridge Program, Fisk University, Nashville, TN USA; 2 Department of Life and Physical Sciences, Fisk University, Nashville, TN USA; 3 Department of Pharmacology, Vanderbilt University, Nashville, TN USA

**Keywords:** Cancer, Cancer-testis antigens, Melanoma antigen genes, Anchorage-independent growth, Epigenetics, DNA methylation, Gene expression, Cell proliferation, Chemo-resistance

## Abstract

Melanoma Antigen Genes (MAGEs) are a family of genes that have piqued the interest of scientists for their unique expression pattern. A subset of MAGEs (Type I) are expressed in spermatogonial cells and in no other somatic tissue, and then re-expressed in many cancers. Type I MAGEs are often referred to as cancer-testis antigens due to this expression pattern, while Type II MAGEs are more ubiquitous in expression. This study determines the cause and consequence of the aberrant expression of the *MAGE-A* subfamily of cancer-testis antigens. We have discovered that *MAGE-A* genes are regulated by DNA methylation, as revealed by treatment with 5-azacytidine, an inhibitor of DNA methyltransferases. Furthermore, bioinformatics analysis of existing methylome sequencing data also corroborates our findings. The consequence of expressing certain *MAGE-A* genes is an increase in cell proliferation and colony formation and resistance to chemo-therapeutic agent 5-fluorouracil and DNA damaging agent sodium arsenite. Taken together, these data indicate that DNA methylation plays a crucial role in regulating the expression of *MAGE-A* genes which then act as drivers of cell proliferation, anchorage-independent growth and chemo-resistance that is critical for cancer-cell survival.

## Introduction

**Fig. 1:**
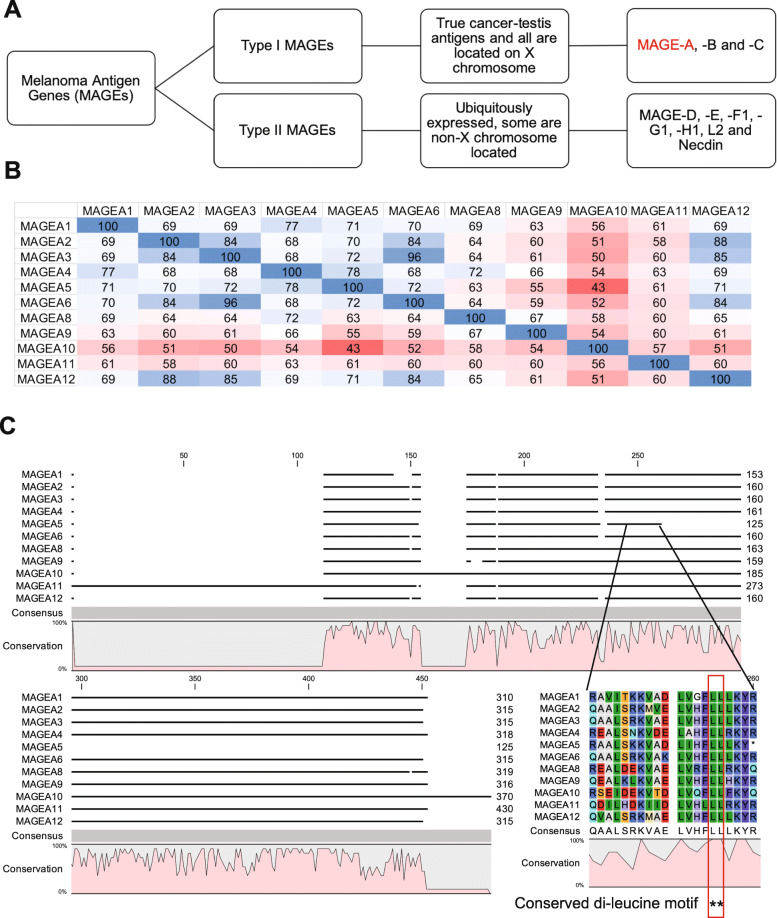
Introduction of *MAGE-A* subfamily of genes and their protein products. **a** Schematic illustrating the focus of this paper. Melanoma Antigen Genes are divided into Type I and Type II based on their expression pattern. *MAGE-A* genes are considered *bona fide* cancer-testis antigens and are located on the X-chromosome. Type II MAGEs are ubiquitously expressed, and all members are not located on the X chromosome. **b** Clustal W sequence alignment shows the different percentages of sequence identity among the MAGE-A proteins. **c** Alignment of individual protein sequences shows that MAGE-A proteins share a MAGE homology domain (pink region) and an invariant dileucine motif (indicated by **)

Melanoma Antigen Genes (MAGEs) were first discovered because a patient with melanoma and a few melanoma cell lines presented an antigen that was recognized by cytotoxic T-cells. Subsequent autologous typing led to the discovery of MAGE-1, now known as MAGEA1, as the tumor associated antigen [1]. Since then, based on sequence similarity, more gene members have been added to the family in humans, with a total of about 60 genes including pseudogenes [2]. MAGEs can be divided into Type I and Type II based on their expression pattern and their chromosomal location (Fig. 1a). Type I MAGEs are all reported to be cancer-testis antigens, and located on the X-chromosome, whereas Type II MAGEs are ubiquitous in expression and some members such as *MAGEL2* are located on autosomes [3]. All MAGE proteins share a MAGE homology domain (MHD) and some members of this enigmatic family bind to E3-ubiquitin ligases and enhance their activity, by as yet unknown mechanisms [4]. This adaptor function of the MAGE proteins results in regulation of many biological processes. For example, MAGEA3/6, a Type I MAGE regulates degradation of AMPK, a master metabolic regulator and tumor-suppressor [5], and activation of cancer-specific MAGEA11-HUWE1 ligase complex leads to alternate polyadenylation of core oncogenic and tumor suppressor transcripts [6], whereas MAGEL2, which is a type II MAGE, regulates protein trafficking by ubiquitination of WASH, a known mediator of the retromer complex [7, 8].

Type I MAGEs have garnered a lot of interest because of their unique expression pattern. As cancer therapy is becoming more personalized, being able to target cancer cells specifically, is attractive. Therefore, genes and their protein products that are exclusively expressed in cancer cells such as the MAGE proteins have good therapeutic potential. However, there is a significant gap in the knowledge of how the expression of each of these genes is regulated and their individual contribution towards the process of either initiation or maintenance of cancer phenotypes. Furthermore, if we target one, do we need to target them all?

Many germline genes are regulated by epigenetic mechanisms, such as promoter methylation, histone methylation, and other post-translational modifications of histones that affects chromatin state [9–11]. In fact, the epigenetic landscape of the spermatozoon is thought to contribute transgenerational epigenetic inheritance [12]. In addition, there is evidence that *MAGEA1* and *MAGEA11* are both regulated by CpG methylation [13–15]. However, what is not clear is whether expression of **all***MAGE-A* genes is regulated by methylation, as would be expected since many *MAGE-A* genes are co-expressed in cancers, and whether aberrant expression of **each** of these genes contributes to the process of cellular transformation in any way. A recent study has shown that in mice, *MAGE-A* genes protect spermatogonial cells from genotoxic stress [16]. The role that these genes, either collectively as a gene family, or individually, play in cancer-associated phenotypes is a little less clear.

This study is focused on *MAGE-A* sub-family of genes and its protein products and the role they play in cancer. This sub-family has garnered interest because MAGEA3 peptides were used in a clinical trial for non-small cell lung cancer, as an immunostimulant and more recently, a study established MAGE-A proteins as predictors of resistance to anti-CTLA4 therapy in patients with metastatic melanoma [17–19]. Sequence alignment of the full-length MAGE-A proteins reveals varying levels of sequence identity. For example, MAGEA3 and MAGEA6 are nearly identical (96% identity) and more similar to MAGEA2 and MAGEA12 (84 and 85% identity), while MAGEA5 is an outlier with a truncated MHD. In addition, MAGEA10 and MAGEA11 at most share only 58 and 63% identity with other MAGEA proteins, respectively (Fig. 1b). Nevertheless, all MAGE-A proteins share a conserved di-leucine motif (Fig. 1c).

We have tested the hypothesis that changes in epigenetic landscape, specifically DNA methylation, a common mechanism that regulates expression of many genes in both cancer and the germ line, regulates expression of *MAGE-A* genes as well, and when expressed *MAGE-A* genes have specific roles to play in the cancer cell. We have discovered that indeed all members of the *MAGE-A* gene family are regulated by DNA methylation. We present evidence that each *MAGE-A* gene might have a different role to play in the process of cellular transformation.

## Results

### *MAGE-A* genes are enriched in the bone marrow, cancer and testis

**Fig. 2:**
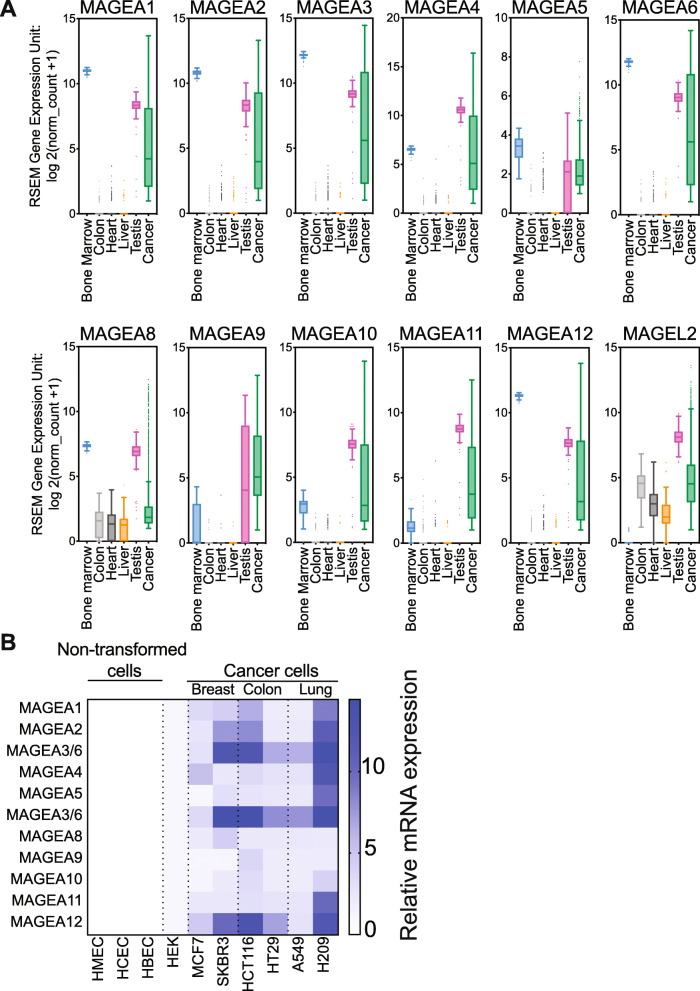
Human *MAGE-A* genes are true cancer testis-antigens. **a** mRNA expression of *MAGE-A* genes from TCGA PAN-Cancer and GTEX datasets in bone marrow (blue), colon (grey), heart (black), liver (orange) and testis (pink). *MAGEL2* is a ubiquitously expressed Type II MAGE. **b** RT-qPCR expression of *MAGE-A* genes in primary, immortalized human mammary, colonic, and bronchial epithelial cells, HEK cells and in patient-derived cancer lines MCF7, SKBR3 (breast cancer), HCT116, HT29 (colon cancer), A549 and H209 (lung cancer)

In order to determine whether all members of the *MAGE-A* gene family strictly adhered to the expression pattern of a cancer-testis antigen, we used UCSC Xena functional genomics browser to analyze mRNA expression of *MAGE-A* genes from RNA-Sequencing datasets from the TCGA (PAN-CANCER) and GTEx dataset for bone marrow, colon, heart, liver and, testis samples [20]. We used these tissues specifically because, the heart is a highly differentiated tissue [21], while colon and liver are differentiated but still proliferative [22, 23] and the bone marrow, which is enriched in stem cells [24]. As a negative control, we also determined the expression pattern of *MAGEL2*, a more ubiquitously expressed Type II MAGE gene [3, 7]. Not surprisingly, *MAGEL2* was expressed in all tissues, except the bone marrow (Fig. 2a). With the exception of *MAGEA8*, we found that all the *MAGE-A* genes, were exclusively expressed in the bone marrow, testis and in cancer tissue (Fig. 2a). Moreover, the level of expression of the *MAGE-A* genes was highly variable within the PAN-CANCER samples. To confirm if this expression profile translates into cell culture models, we next performed RT-qPCR analysis on normal and cancer cell lines. We determined the relative mRNA levels of *MAGE-A* genes in normal non-transformed cell lines from three different tissue origins, breast (Human Mammary Epithelial Cells, HMEC), lung (Human Bronchial Epithelial Cells, HBEC), and colon (Human Colonic Epithelial Cells, HCEC). We detected no expression of *MAGE-A* genes in these non-transformed cell lines (Fig. 2b). HEK 293 cells have either undetectable or low basal expression of a few *MAGE-A* genes, however patient derived cancer cell lines such as SKBR3 and MCF7 (breast cancer), HCT116 and HT29 (colon cancer), A549 and H209 (lung cancer) cells lines expressed high levels of most of the *MAGE-A* genes (Fig. 2b). Within cell lines of each type of cancer, SKBR3, HCT116 and H209 displayed higher expression of *MAGE-A* genes. Similarly, expression of *MAGEA3*, *MAGEA6* and *MAGEA12* was greater than 3-fold in all cancer lines, regardless of origin, when compared to HEK cells. Taken together, these data indicate that *MAGE-A* genes are not expressed in any normal tissue or cell lines, but highly expressed in cancer cells, testis and the bone marrow. These data suggest that there might be similarities in the epigenetic landscape of the gene regulatory elements controlling expression of *MAGE-A* genes in these tissues.

### DNA-methylation regulates expression of *MAGEA* genes

**Fig. 3:**
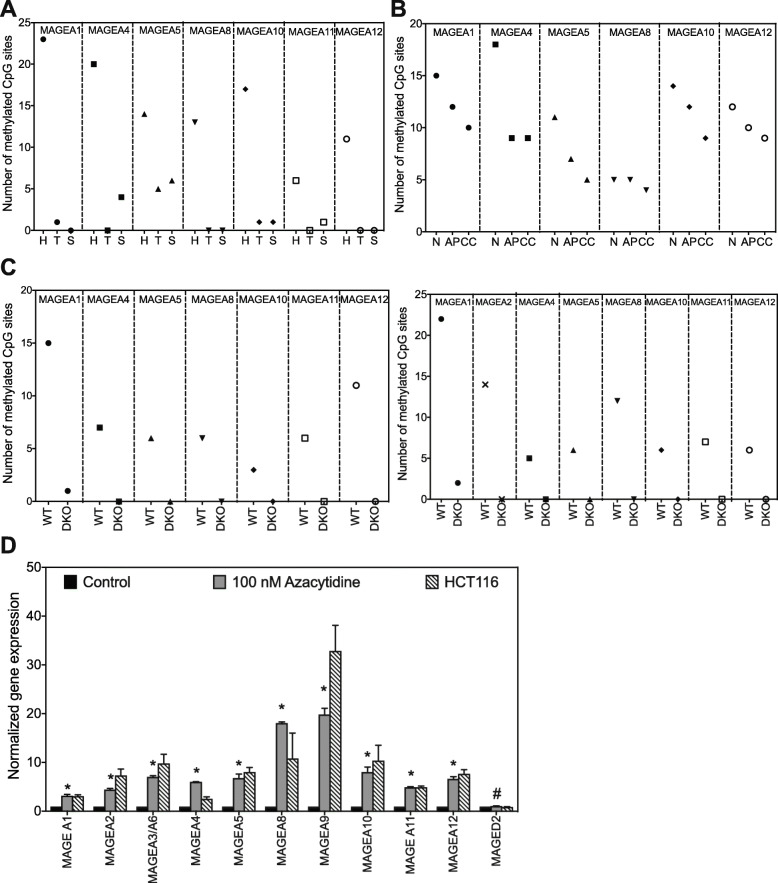
Expression of *MAGE-A* genes correlates with hypomethylation of promoter regions. **a** Number of fully methylated CpG sites 1000 bp upstream of transcription start site of each *MAGE -A *gene were counted using methylome datasets in the UCSC genome browser in the indicated tissue (H) heart, (T) testis and (S) Sperm. **b** Number of fully methylated CpG sites 1000 bp upstream of transcription start site of each *MAGE -A* gene were counted using methylome datasets in the UCSC genome browser in the indicated tissue, (N) normal colon, (AP) adenomatous polyp, (CC) colon cancer tissue. **c** Number of fully methylated CpG sites 1000 bp upstream of transcription start site of each *MAGE -A* gene were counted using two methylome datasets in the UCSC genome browser in HCT116 wild type cell line (WT) and HCT116 where DNMT1 and DNMT3b (DNA methyl transferases) have been depleted (DKO). **d** mRNA expression of the indicated *MAGE* gene in HEK cells treated with DMSO (black) or 100 nM Azacytidine (grey) or HCT116 cancer cell line (lined). Expression of each MAGE gene is normalized to expression in HEK cells. Data shown are mean ± S.D. from n = 3 experiments. *P* values were determined from Students' t-test and * *p* < 0.001 and # is not-significant (NS)

Cancer-testis antigens, including certain MAGE genes, XAGE1, NY-ESO1 have been found to be regulated by DNA methylation [19, 25, 26]. Since both cancer tissue and cell lines have coordinated high expression of *several MAGE-A* mRNA, and yet exhibit some heterogeneity in expression levels (Fig. 2b), we sought to confirm that DNA methylation plays a universal role in regulating expression of *MAGE-A* genes. We first performed bioinformatics analysis using the UCSC Genome Browser and determined the number of methylated CpG sites 1000 bp upstream of transcription start sites of *MAGE-A* genes in tissues where *MAGE-A* expression is high versus low or non-existent. Consistent with previous data and our hypothesis, there is a distinct pattern of hypomethylation in tissues where *MAGE-A* genes are expressed such as testis and sperm [27, 28] compared to heart where *MAGE-A* genes are not expressed [29] (Fig. 3a). We then analyzed methylation of *MAGE-A* promoters in normal colon, adenomatous polyp and colon cancer tissue [30]. We determined that in colon cancer tissue there was a concomitant decrease in methylation of *MAGE-A* promoters when compared to that of normal colon mucosa or adenomatous polyp (Fig. 3b). To further our bioinformatics study, we determined the number of methylated CpG in patient-derived cancer line HCT116 (WT) versus HCT116 cells with a double knock-out for DNA-methyltransferases (DNMTs) *DNMT1* and *DNMT3b.* As shown in Fig. 3c, in two independent studies [31, 32], the number of methylated cytosines decreases in *MAGE-A* promoters when DNA-methyltransferases (DNMT1 and DNMT3b) are depleted.

To complement our bioinformatics approach, we used a DNMT inhibitor 5-azacytidine [33] to ascertain whether prolonged and low dose treatment could elicit *MAGE-A* expression. Indeed, expression of several *MAGE-A* genes increased in HEK cells treated with 100 nM 5-azacytidine, in some cases almost to levels comparable to HCT116 cancer cell line (Fig. 3d). The expression of *MAGED2,* a ubiquitously expressed Type II MAGE did not change with 5-azacytidine exposure. Our data confirm that *MAGE-A* genes are indeed regulated by DNA methylation of proximal promoter regions.

### *MAGE-A* genes increase proliferation and anchorage-independent growth of cells

**Fig. 4:**
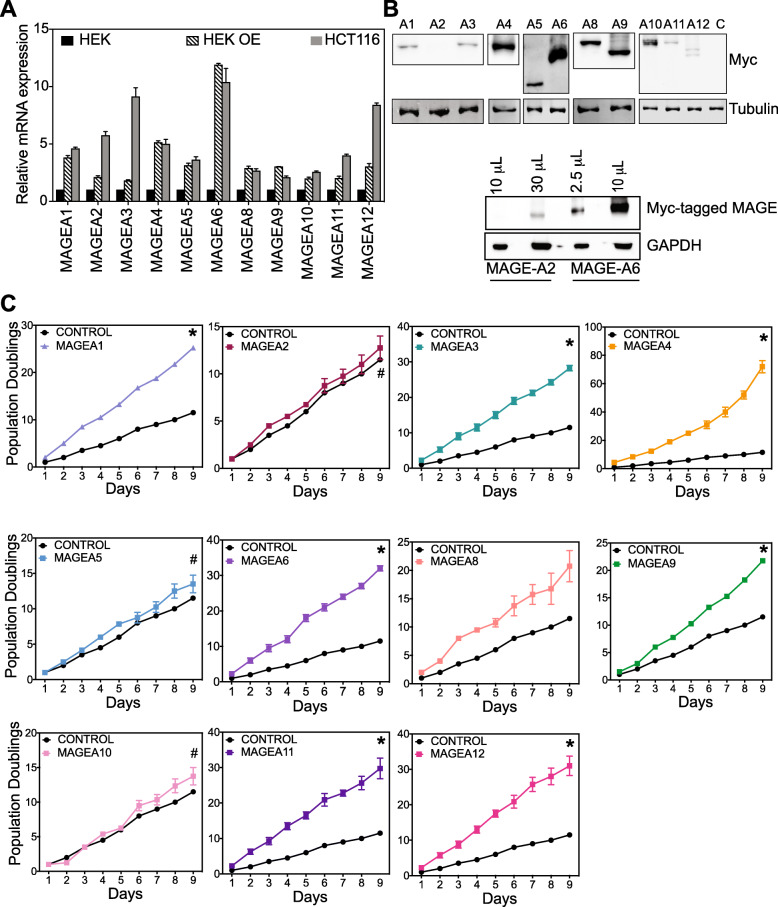
Cells expressing MAGE-A genes have a proliferative advantage. **a** mRNA expression *MAGE-A* genes in HEK (black), HEK OE (HEK cells that have been transduced with lentivirus encoding the indicated MAGE gene, lined), HCT116 (grey) as determined by RT-qPCR. Data shown are mean ± S.D. from n = 3 experiments. **b** Western blot showing expression of myc-tagged MAGE-A proteins in the lysates of HEK OE cell lines. Tubulin is used as loading control. **c** Population doubling of HEK cells or HEK cells expressing specific and indicated MAGEs over a period of 9 days. Cells were counted every 24 hours to determine the population doubling. Data shown are mean ± SEM from n = 3 experiments. *P* values were determined from Students' t-test and * *p* < 0.01 and # is not-significant (NS)

Global DNA methylation changes leading to dysregulation of many genes is common to cancer and other physiological conditions such as aging [34], but the question we sought to answer was; were the *MAGE-A* genes all interchangeable, or does each gene and protein product have a distinct role to play in cellular transformation. Therefore, to ascertain the consequence of aberrant methylation-driven expression of these cancer-testis antigens, we over-expressed all *MAGE-A* genes in HEK cells using a lentiviral expression system. To make sure that any phenotypes we obtain were not due to artifactual over-expression effects, we first compared the mRNA levels of each *MAGE-A* gene in our over-expression cell lines to the expression in patient-derived HCT116 cancer cell line*. MAGEA1, −A4, −A5, −A6, −A8, −A9, − 10* were all expressed at the same level as that of cancer cell lines. *MAGEA2, −A3, −A11 and -A12* had lower expression than cancer lines but higher compared to our vector control HEK cells (Fig. 4a). To determine whether the over-expression translated to protein levels, we performed western blot analyses of lysates. All the MAGE-A proteins were detected, and protein expression correlated with mRNA levels (Fig. 4b).

**Fig. 5:**
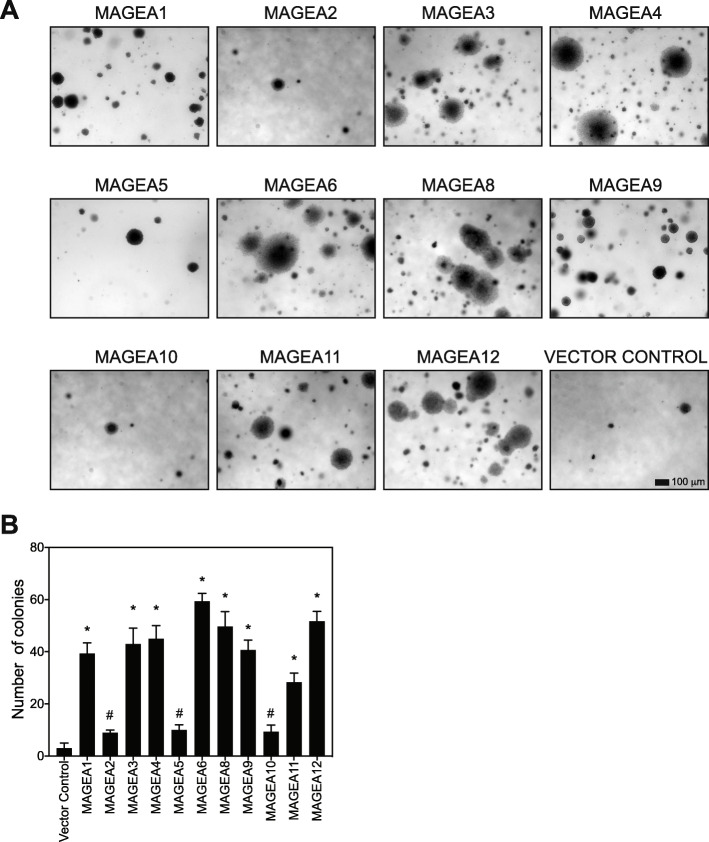
Cells expressing MAGE-A genes display increased anchorageindependent growth. **a** and **b** HEK cells expressing vector control or indicated MAGEs were plated in soft agar colony formation assay and colonies were imaged and counted on day 15. Data shown are mean ± S.D. from *n* = 3 experiments. *P* values were determined from Students' t-test and * *p* < 0.01 and # is not-significant (NS)

To measure proliferation rates, we determined the number of population doublings of each cell line over a period of 9 days. MAGEA1, −A3, −A4, −A6, −A8, −A9, −A11 and -A12 expressing cell lines showed increased proliferation compared to cells expressing vector control, while MAGEA2, −A5 and -A10 expressing cells did not (Fig. 4c). Anchorage-independent growth is considered a hallmark of cancer [35, 36], therefore we determined the ability of MAGE-A expressing cells to grow colonies in soft agar (Fig. 5). MAGEA3, −A4, −A6, −A8, A11 and -A12 expressing cells displayed the ability to form larger colonies (Fig. 5a) and MAGEA1, −A3, −A4, −A6, −A8, −A9, −A11 and -A12 expressing cells showed over 3-fold increase in number of colonies compared to vector control cells, while MAGEA2,-A5 and -A10 do not (Fig. 5b). Taken together with proliferative capacity of these cells, our data indicate that several members of the *MAGE-A* family act as drivers of cancer phenotypes.

### Expression of *MAGE-A* genes results in protection from chemical stressors

**Fig. 6:**
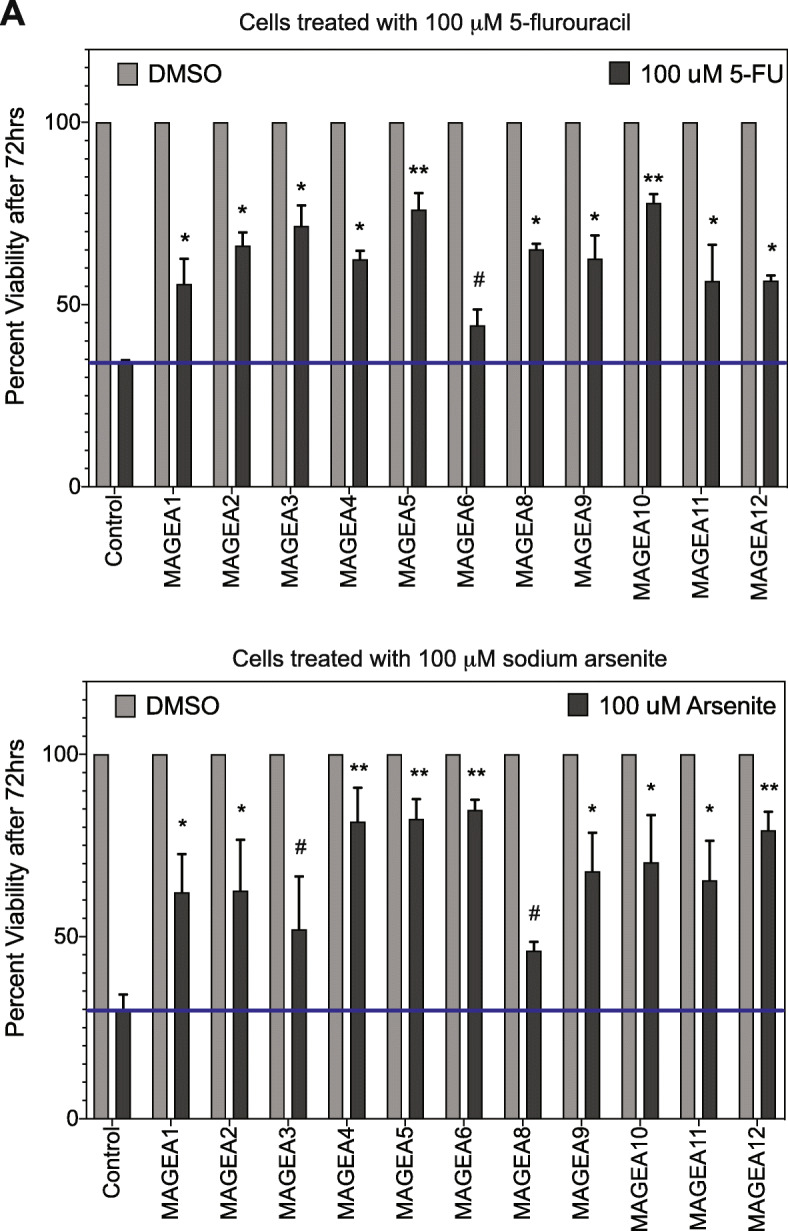
Specific MAGE-A genes confer chemo-resistance. **a** HEK cells expressing vector control or MAGE-A genes were treated with 100 μM 5-fluorouracil and cell viability measured at 72 hours post-treatment using the end point Cell-Titer Glo assay. The luminescence of cells treated with DMSO was set to 100% viability. Data shown are mean ± S.D. from n = 3 experiments. *P* values were determined from Students' t-test and * *p* < 0.01, ** *p* <0.001 and # is not significant. **b** HEK cells expressing vector control or MAGE-A genes were treated with 100 μM sodium arsenite and cell viability measured at 72 hours post-treatment using the end point Cell-Titer Glo assay. The luminescence of cells treated with DMSO was set to 100% viability. Data shown are mean ± S.D. from n = 3 experiments. *P* values were determined from Students' t-test and * *p* < 0.01

Several studies have shown that expression of certain members of the *MAGE-A* family results in poor patient prognoses [5, 19, 37] and being able to parse out treatment strategies based on MAGE expression might be critical. In order to determine whether MAGE-A expressing cells confer protection against chemotherapeutic or DNA-damaging agents, and therefore allow for cancer persistence and poor prognoses, we treated control or MAGE-A expressing cells to high dose (100 μM) of 5-fluorouracil or sodium arsenite. MAGE-expressing cells are more viable with high doses of chemotherapeutic agent 5-Fluorouracil or DNA-damaging agent sodium arsenite compared to control cells, but MAGE-A5 and MAGE-A10 confer almost 80% survival to these cells (Fig. 6a and b). Our data indicate that aberrant expression of MAGE-A proteins in cells allows for cell survival even in the presence of extraneous stressors.

## Discussion

There are many hallmarks of cancer tissue, genomic instability and general deregulation of expression of many genes is one of them [38, 39]. Melanoma Antigen Genes (MAGEs) are interesting because of their restricted expression pattern, in the male germline and in many cancers (Fig. 2). This expression pattern combined with the fact that the testis is an immune-privileged tissue [40], makes the MAGE-A proteins attractive targets for cancer immuno-therapy as well as for directed drug design. The main conundrum in cancer biology is to determine if genomic changes in tumors represent changes in expression of genes and their protein products causal for disease initiation and progression, and thus make for rational therapeutic targets, or are they simply a manifestation of overall dysfunction in gene expression with no causal disease impact. Determining whether or not, certain MAGEs are causal for disease-related phenomena in cancer, is the basis for these studies.

In this study, we sought out to determine whether methylation plays a role in reactivating the expression of these genes in cancer. Our study, along with bioinformatics analysis of existing methylome sequencing databases confirms that indeed DNA methylation does regulate the expression of this *MAGE-A* subfamily of genes. Furthermore, once expressed, the next big question to be answered is what are the consequences of *MAGE-A* expression? Some of the individual MAGEs have been studied in detail [4–6, 41], but whether each MAGE-A protein contributes to cellular transformation and whether some MAGE-A proteins are interchangeable with the others, and are they all activated at the same time or some are activated early and some later in the process of transformation is unclear. Our studies here have begun to parse out the differences in efficacy of each MAGE-A protein in contributing to cellular transformation (Figs. 4, 5 and 6). Our data indicate that not all MAGE-A proteins cause similar phenotypes. For example, MAGEA5 and MAGEA10 are not associated with increased population doubling or anchorage-independent growth, but considerably rescue decrease in cell viability upon treatment with high dose of 5-flurouracil (Fig. 6a). This is an interesting and rather surprising finding. As shown in Fig. 1b and c, MAGEA5 and MAGEA10 share only 43% sequence identity. However, MAGEA5 retains the di-leucine motif that is found in all MAGE proteins (Fig. 1b) and thought to be important for biochemical function [4]. Interestingly, there exists a *MAGEA5-MAGEA10* read-through transcript or conjoined gene [42, 43] and we speculate whether expression of the MAGEA5 or MAGEA10 protein in HEK cells that do not express any MAGEA5 or MAGEA10 (Fig. 2b) mimics expression of the read-through transcript and its gene product in cancer, and whether this would explain why MAGEA5 and MAGEA10 have the same effect on cellular homeostasis.

Our data indicate that there is no concentration or dose-dependence correlations between MAGE-A expression and phenotypes. For example, MAGEA12 is expressed at very low levels compared to MAGEA6 and yet both are capable of eliciting cell proliferation and anchorage-independent growth. This interpretation is in keeping with the expression pattern of these genes between cancer and non-tumorigenic tissue normal cells, which is all or nothing.

The fact that the *MAGE-A* genes play a similar role in both spermatogonial cells [16] and cancer cells might suggest that an epigenetic program is reactivated by memory and it would be interesting to determine if all cells within the heterogenous tumor express *MAGE-A* genes or there exists a specific subset of cells that express these genes. It might also be of interest to perform detailed analysis of the epigenetic landscapes of MAGE gene regulatory regions, including *cis-acting* factors such as enhancers, in all the tissues they are expressed, such as the testis, cancer cells, bone marrow and placenta (Fig. 2 and [16]).Our data seem to suggest that based on the pleotropic effects of different MAGEs they are activated at different times and most importantly, sequencing and or proteomic profiling of tumors for *MAGE-A* genes and their protein products is necessary before therapies can be designed. In addition, analyzing the signaling mechanisms by which each MAGE-A protein causes increase in cell proliferation or anchorage-independent growth might give clues regarding pathways or proteins to target.

## Conclusions

In summary, our studies show that *MAGE-A* expression and reactivation in cancer is caused by changes in DNA methylation and that the consequence of this aberrant expression is that it allows to cells to proliferate faster, change their anchorage-dependence and survive in the presence of high doses of chemotherapeutic drugs. This is the first step in understanding the role that each MAGE-A protein plays in cellular transformation.

## Materials and method

### Cell lines and culture

Human Colonic epithelial line (HCEC-CT) was cultured and maintained as described in [5, 44]. Human bronchial epithelial cells (HBEC3-KT) were cultured and maintained at described in [45]. Human mammary epithelial cells were purchased from ATCC (PCS600–010) and maintained in mammary epithelial cell medium supplemented with mammary epithelial cell growth kit (ATCC, PCS600–030 and PCS600–040). HEK293 cells were a kind gift from Dr. Shawn Goodwin at Meharry Medical College and were maintained in DMEM with 10% fetal bovine serum (FBS) and antibiotics. 293FT cells were purchased from Thermo Fisher (R70007) and were cultured in DMEM with 10% FBS and antibiotics. MCF7 and SKBR3 cells were a kind gift from Dr. Ann Richmond at Vanderbilt University and were grown in DMEMF12/Ham media with 10% FBS and antibiotics. HCT116, HT29 and A549 cells were grown in DMEM with 10% FBS and antibiotics. H209 (ATCC, HTB-172) cells were grown in RPMI with 10% FBS and antibiotics. All cells were grown at 37 °C with 5% CO_2_ unless otherwise indicated. Source of cell lines and citations are shown in Table 1. Cells were counted either using a traditional hemocytometer or Bio-Rad (TC20™ Automated Cell counter). Slides were purchased from Vanderbilt Molecular Biology Core.

**Table 1:** Cell lines used

Cell line	Phenotype	Immortalization	Origin/Reference
HMEC (human mammary epithelial cells)	Normal, primary, non-tumorigenic	N/A	ATCC PCS600–010
HCEC-CT (human colonic epithelial cells)	Normal, non-tumorigenic	CDK4, hTERT	[5, 44]
HBEC3-KT (human bronchial epithelial cells)	Normal, non-tumorigenic	CDK4, hTERT	[45]
HEK293	Tumorigenic only at high passages	Ad5	ATCC/Goodwin lab at Meharry
MCF7	Cancerous, human patient derived breast adenocarcinoma	N/A	ATCC HTB-22/Richmond lab
SKBR3	Cancerous, human patient derived breast adenocarcinoma	N/A	ATCC HTB-30/Richmond lab
HCT116	Cancerous, human colorectal carcinoma	N/A	ATCC CCL-247
HT29	Cancerous, human colorectal adenocarcinoma	N/A	ATCC HTB-38
A549	Cancerous, lung carcinoma	N/A	ATCC CCL-185
H209	Cancerous, lung, derived from metastatic bone marrow	N/A	ATCC HTB-172

### Bioinformatics

### UCSC Xena platform for *MAGE-A* expression profile

GTEx and TCGA PAN-Cancer expression datasets for each of the *MAGEA* genes were downloaded. Heart tissue is used as a representative of normal somatic tissue. Of the TCGA Pan-Cancer dataset, only those cancer tissue samples in which *MAGEA* mRNA levels were greater than two-fold in expression were represented in the histogram.

### UCSC genome browser for methylome profile

Using existing methylome sequencing data published in the UCSC Genome browser, we analyzed and counted the methylated CpG sites 1000 bp upstream of each *MAGEA* gene in each of the respective studies [28]. study was used for the heart, testis and sperm methylome data [30]. was used for the normal, adenomatous polyp and colon cancer data and [31, 32] was used for the HCT116 versus HCT116 with double knock-out for DNA methyltransferases (DNMT1 and DNMT3b).

#### Azacytidine assay

Indicated cells were maintained in media containing DMSO control or 100 nM 5-azacytidine (Sigma Aldrich, A2385) for one week at which point cells were harvested and expression of MAGE mRNA was determined by RT-qPCR.

#### RT-qPCR

RNA from indicated cell lines and treatment conditions was purified using RNA-easy kit (Qiagen, 74104) using standard manufacturer’s protocol. Following DNAse (Life Technologies, 18068015) treatment, 300 ng of RNA from every experimental condition was used for a one-step reverse-transcription and quantitative PCR using BioRad CFX Maestro thermocycler iQ Sybr Green Master mix (Biorad, 1708880). Primer sequences for all MAGE genes and housekeeping genes are given in Table 2.

**Table 2:** RT qPCR primers

Gene	Forward primer	Reverse primer
MAGEA1	caacccagaggacaggattc	gggcaatgaagacccaca
MAGEA2	atgaacgggctttgagagag	gaggcagtggaagctaatgg
MAGEA3/A6	gtgaggaggcaaggttctga	gggcaatggagacccact
MAGEA4	acagaggagcaccaaggaga	cagcaggcaagagtgcag
MAGEA5	agaggagcaccaaaggagaa	actctggtcaccgcaacag
MAGEA8	cactcctacatccttgtcacctg	ggtcttgggcgtactctgat
MAGEA9	ggagaggcctccttctgag	tctgcgacctgaggacact
MAGEA10	gttctgagggacaggcttga	gtcaccctctgagagcaagg
MAGEA11	tctttctgaggggtgtcttgag	gaactgagtctccatccctcag
MAGEA12	gattctcgccctgagcaa	gggcctgtctcctcagaac
MAGEA1 (CDS)	gagtccttgttccgagcagt	aggagcagaaaaccaaccaa
MAGEA2 (CDS)	caatagagggcgactgtgc	ccctcaaacacctccaacat
MAGEA3/A6 (CDS)	aaggtggccaagttggttc	gcatttctgcctttgtgacc
MAGEA4 (CDS)	cgcctctgaggaggaaatct	ccaatcttgggtgagcagtt
MAGEA9 (CDS)	ttcatgcaggtgatctttgg	acgagaggccaagagcagt
MAGEA10 (CDS)	gccactcctttgtccttgtc	gggcatgctctggacatc
MAGEA11 (CDS)	gtgggtgcacaggctctc	gcccacattcagagtagagga
MAGEA12 (CDS)	ctctctggagtcaatccgatg	caggtcaggaaaggtgcttg
MAGED2	ccagcaagatgaaagtcctca	tccatcgcctctcggtact
RpLp (housekeeping gene)	tctacaaccctgaagtgcttgat	caatctgcagacagacactgg

#### Lentiviral expression of MAGE proteins in HEK cells

Lentivirus encoding each MAGE gene was generated by transfecting 1 μg of pLenti6-blast plasmid encoding myc-tagged MAGE gene along with 0.5 μg of pSPAX and pMD2.G plasmids (Addgene) into 293FT cells. 48-h and 72-h supernatant containing the lentiviruses were combined, aliquoted and stored at − 80 °C. The viral supernatants were then added to HEK cells along with polybrene (Sigma Aldrich, TR1003-G), such that multiplicity of infection was = 5. Viral titers were determined used p24 ELISA kit (Xpress Bio-XB1000). 48 h later cells were moved to media containing blasticidin. Expression of individual MAGEs was tested both by RT-qPCR and western blotting. For western blotting, cells were lysed in RIPA buffer (50 mM Tris-HCl, 150 mM NaCl, pH 7.5, 0.5% NP-40) and protein content quantified using BCA Assay (Pierce™ BCA protein assay, 23,225). 10 μg of total protein (at 1 μg/ μL) was loaded for the initial western. For the follow-up western to make sure MAGEA2 was expressed, the indicated volumes of freshly generated lysates were loaded per lane (at 1 μg/ μL). Anti-myc antibody (9E10) was purchased at the Vanderbilt Molecular Biology Core (VAPRE9E10) and used at a 1:1000 dilution and anti-GAPDH antibody was purchased from Santa Cruz Biotech (sc-47724) and used at a 1: 5000 dilution. Anti-mouse IgG conjugated to HRP was purchased from Vanderbilt Molecular Biology Core (Promega-W4021) and was used at a dilution of 1:10000.

#### Phenotypic assays

##### Population doubling

Five thousand control cells or MAGE-expressing cells were plated in replicates in 6-well dishes and counted using a hemocytometer and automated cell counter every 24 h to determine population doubling times for a period of 9 days. Error bars indicate mean and standard deviation from n = 3 measurements.

##### Anchorage-independent growth

A 0.5% agar (Difco Noble Agar from BD Biosciences) base layer was made in each well of a 6-well dish by dissolving 1 g of agar in autoclaved dH_2_O and then mixing the heated agar solution 1:1 with serum-free DMEM. After this layer had solidified, a 0.375% agar cell layer was made by dissolving 0.75 g of agar in autoclaved dH_2_O and then mixing the heated agar solution 1:1 with serum free DMEM. 100,000 control or MAGE-expressing cells that were trypsinized to ensure single cell suspensions were added to this agar solution and this mixture was plated on top of the base layer. Regular media was added on top and media was changed every two days. Colonies > 100 μm were counted at day 15 using a light microscope. Error bars indicate mean and standard deviation from n = 3 measurements.

##### Drug resistance assays

Control cells or MAGE-expressing cells were treated with indicated concentrations of 5-Fluorouracil (5-FU, Sigma Aldrich F6627) or Sodium Arsenite (Sigma Aldrich, S7400) for 72 h and viability was measured using Cell-titer Glo (Promega G7570) on a Tecan luminescence plate reader. Viability at each time point was normalized to luminescence of DMSO control. Error bars indicate mean and standard deviation from n = 3 measurements.
